# Anaphylaxis

**DOI:** 10.1186/s13223-018-0283-4

**Published:** 2018-09-12

**Authors:** David Fischer, Timothy K. Vander Leek, Anne K. Ellis, Harold Kim

**Affiliations:** 1 Western University, London, ON Canada; 2 University of Alberta, Edmonton, AB Canada; 3 Queen’s University, Kingston, ON Canada; 4 McMaster University, Hamilton, ON Canada

**Keywords:** Anaphylaxis, Diagnosis, Acute management, Epinephrine, Long-term management, Anaphylaxis action plan

## Abstract

Anaphylaxis is an acute, potentially fatal systemic allergic reaction with varied mechanisms and clinical presentations. Although prompt recognition and treatment of anaphylaxis are imperative, both patients and healthcare professionals often fail to recognize and diagnose early signs and symptoms of the condition. Clinical manifestations vary widely; however, the most common signs are cutaneous symptoms, including urticaria, angioedema, erythema and pruritus. Immediate intramuscular administration of epinephrine into the anterolateral thigh is first-line therapy, even if the diagnosis is uncertain. The mainstays of long-term management include specialist assessment, avoidance measures, and the provision of an epinephrine auto-injector and an individualized anaphylaxis action plan. This article provides an overview of the causes, clinical features, diagnosis and acute and long-term management of this serious allergic reaction.

## Background

Anaphylaxis is the most severe form of an allergic reaction—it is rapid in onset and potentially fatal. While the prevalence of anaphylaxis is estimated to be as high as 2% and appears to be increasing, the fatality rate is extremely low (i.e., < 0.0001% prevalence in the general population, or < 0.5% case fatality rate in those hospitalized or presenting to the emergency department with anaphylaxis) and appears to be decreasing [1–3]. Nonetheless, it is essential to be vigilant for those at risk of anaphylaxis, and to establish appropriate measures with the goal of reducing the risk of death from anaphylaxis even further.

Prompt recognition and management of anaphylaxis are imperative. However, the condition is often under-recognized and treated inadequately. Diagnosis and management are challenging since reactions are often immediate and unexpected. Furthermore, there is no single test to diagnose anaphylaxis in routine clinical practice. This article will provide an overview of the causes and clinical features of anaphylaxis as well as strategies for the accurate diagnosis and management of the condition.

## Causes

Most episodes of anaphylaxis are triggered through an immunologic mechanism involving immunoglobulin E (IgE) which leads to mast cell and basophil activation and the subsequent release of inflammatory mediators such as histamine, platelet activating factor, leukotrienes, tryptase and prostaglandins. Although any substance has the potential to cause anaphylaxis, the most common causes of IgE-mediated anaphylaxis are foods (particularly peanuts, tree nuts, shellfish and fish, cow’s milk, eggs and wheat), medications (most commonly penicillin and other antibiotics), and stinging insects [4]. Exercise, aspirin, non-steroidal anti-inflammatory drugs (NSAID), opiates, and radiocontrast agents can also cause anaphylaxis, but anaphylactic reactions to these agents often result from non-IgE-mediated mechanisms. In other cases, the cause of anaphylactic reactions is unknown (idiopathic anaphylaxis). In children, anaphylaxis is most often caused by foods, while venom- and drug-induced anaphylaxis is more common in adults [5–8]. Table 1 provides a more comprehensive list of the potential causes of anaphylaxis.

**Table 1:** Causes of anaphylaxis

**Common**
• Foods: most commonly peanuts, tree nuts, egg, fish, shellfish, cow’s milk, and wheat
• Medications: most commonly antibiotics and NSAID
• Allergen immunotherapy
• Insect stings (bees and wasps)
• Unidentified (no cause found; idiopathic anaphylaxis)
**Less common**
• Exercise
• Natural rubber latex
• Semen
• Hormonal changes: menstrual factors
• Topical medications (e.g., chlorhexidine, polysporin)
• Transfusions

## Co-morbidities

Co-morbidities and medications may also affect the severity of anaphylactic reactions and patient response to treatment. For example, patients with asthma and cardiovascular disease are more likely to experience a poor outcome from anaphylaxis. Concurrent administration of beta-blockers can interfere with the patient’s ability to respond to epinephrine, the first-line of treatment for anaphylaxis (discussed later). Additionally, the use of angiotensin-converting enzyme (ACE) inhibitors may impact a patient’s compensatory physiologic response to anaphylaxis, leading to more severe reactions, although evidence is conflicting [4, 5]. In fact, recent evidence suggests that the use of any antihypertensive medication may worsen an anaphylactic reaction [9].

## Signs and symptoms

As anaphylaxis is a generalized systemic reaction, a wide variety of clinical signs and symptoms involving the skin, gastrointestinal and respiratory tracts, and cardiovascular system can be observed (see Table 2). The most common clinical manifestations are cutaneous symptoms, including urticaria and angioedema, erythema (flushing), and pruritus (itching) [10]. Patients also often describe an impending sense of death (*angor animi*). Death due to anaphylaxis usually occurs as a result of respiratory obstruction or cardiovascular collapse, or both. It is important to note that the signs and symptoms of anaphylaxis are unpredictable and may vary from patient to patient and from one reaction to another. Therefore, the absence of one or more of the common symptoms listed in Table 2 does not rule out anaphylaxis, and should not delay immediate treatment.

**Table 2:** Signs and symptoms of anaphylaxis [10, 16]

**Skin** • Urticaria • Angioedema • Erythema (flushing) • Pruritus • Eczema **Respiratory** • *Upper airway* Nasal congestion Sneezing Hoarseness Cough Laryngeal edema • *Lower airway* Dyspnea Cough Bronchospasm Wheezing Chest tightness **Cardiovascular** • Hypotension • Dizziness • Syncope • Tachycardia	**Gastrointestinal** • Nausea • Vomiting • Abdominal pain • Diarrhea **Neurologic** • Lightheadedness • Dizziness • Confusion **Oropharyngeal** • Pruritus, tingling • Angioedema **Other** • Sense of impending doom • Anxiety

The signs and symptoms of anaphylaxis typically develop within minutes after exposure to the offending antigen, but may occasionally occur as late as 1 h post exposure. Symptoms usually follow a uniphasic course, with resolution of symptoms within hours of treatment. However, between 0.4 and 15% of reactions follow a biphasic course [11] characterized by an asymptomatic period of several hours (1–36 h; mean of 10 h in one case series) [12] followed by recurrent symptoms.

## Diagnosis

The diagnosis of anaphylaxis during an acute episode is based primarily on clinical signs and symptoms. Following the acute episode, confirmation of the diagnosis requires a detailed description of the acute episode, including antecedent activities and events. Diagnostic criteria for anaphylaxis were published by a multidisciplinary group of experts in 2005 and 2006, and are shown in Table 3 [13, 14]. Since confirming the diagnosis and etiology of anaphylaxis is often complex, referral to an allergist with training and expertise in the identification and management of anaphylaxis is strongly encouraged.

**Table 3:** Clinical criteria for diagnosing anaphylaxis [13, 14]

Anaphylaxis is highly likely when any 1 of the following 3 criteria is fulfilled following exposure to an allergen	
1	Acute onset of an illness (minutes to several hours) with involvement of the skin, mucosal tissue, or both (e.g., generalized hives, pruritus or flushing, swollen lips-tongue-uvula) and at least 1 of the following a.** Respiratory compromise** (e.g. dyspnea, wheeze, bronchospasm, stridor, reduced PEF, hypoxemia) b.** Reduced BP** or associated symptoms of end-organ dysfunction (e.g. hypotonia [collapse], syncope, incontinence)
2	Two or more of the following that occur rapidly after exposure to a ***likely*** allergen for that patient (minutes to several hours) a. **Involvement of the skin-mucosal tissue** (e.g., generalized hives, itch-flush, swollen lips-tongue-uvula) b. **Respiratory compromise** (e.g., dyspnea, wheeze, bronchospasm, stridor, reduced PEF, hypoxemia) c. **Reduced BP** or associated symptoms (e.g., hypotonia [collapse], syncope, incontinence) d. **Persistent GI symptoms** (e.g., painful abdominal cramps, vomiting)
3	Reduced BP after exposure to a ***known*** allergen for that patient (minutes to several hours) a. **Infants and children**: low systolic BP (age specific) or > 30% decrease in systolic BP^a^ b. **Adults:** systolic BP < 90 mmHg or > 30% decrease from that person’s baseline

*PEF* peak expiratory flow, *BP* blood pressure, *GI* gastrointestinal

^a^Low systolic blood pressure for children is age specific and defined as: < 70 mmHg for age 1 month to 1 year; < 70 mmHg + [2 × age] for age 1–10 years; < 90 mmHg for age 11–17 years

### History

The clinical history is the most important tool to establish the cause of anaphylaxis and must take precedence over diagnostic tests. It should elicit information about clinical manifestations (e.g., urticaria, angioedema, flushing, pruritus, airway obstruction, gastrointestinal symptoms, syncope, and hypotension), agents encountered immediately prior to the onset of the reaction (e.g. foods, medications or insect bites/stings), as well as the patient’s activities preceding the event (e.g., exercise, sexual activity). The absence of cutaneous symptoms puts the diagnosis in question, since the majority of anaphylactic episodes include cutaneous symptoms; however, their absence does not rule out anaphylaxis [5].

### Diagnostic tests

The diagnosis of a specific cause of anaphylaxis may be supported by the results of skin tests and/or in vitro IgE tests [5]. These tests can determine the presence of specific IgE antibodies to foods, medications (e.g., penicillin), and stinging insects. However, for the majority of medications, standardized skin tests and/or in vitro tests are not available.

The clinical diagnosis of anaphylaxis can sometimes be supported by the documentation of elevated concentrations of mast cell and basophil mediators such as plasma histamine or serum or plasma total tryptase. However, it is critical to obtain blood samples for these measurements as soon as possible after the onset of symptoms since elevations are transient.

### Differential diagnosis

Other diagnoses that might present with signs and/or symptoms characteristic of anaphylaxis should be excluded. The most common conditions that mimic anaphylaxis include: vasovagal reactions (characterized by hypotension, pallor, bradycardia, weakness, nausea and vomiting), vocal cord dysfunction, severe acute asthma, foreign body aspiration, pulmonary embolism, acute anxiety (e.g., panic attack or hyperventilation syndrome), myocardial dysfunction, acute poisoning, hypoglycemia, and seizure [5, 15]. Recurrent episodes of anaphylaxis may suggest underlying systemic mastocytosis.

## Treatment

### Acute management

The acute treatment of anaphylaxis begins with a rapid assessment of circulation and breathing, followed by the immediate administration of epinephrine. Epinephrine is the drug of choice for anaphylaxis and should be given immediately to any patient with suspected anaphylaxis. Treatment should be provided even if the diagnosis is uncertain since there is no contraindication to the use of epinephrine [16].

The recommended dose of epinephrine for anaphylaxis is 0.01 mg/kg (maximum 0.5 mg) administered intramuscularly every 5–15 min as necessary [5, 17]. Intramuscular administration into the anterolateral thigh is recommended as it allows for more rapid absorption and higher plasma epinephrine levels compared to subcutaneous or intramuscular administration in the upper arm [18, 19]. Glucagon should also be considered in patients using beta-blockers.

All patients receiving emergency epinephrine must be transported to hospital immediately (ideally by ambulance) for evaluation and observation. Ideally, patients should be placed in a recumbent (supine) position, unless the respiratory compromise contraindicates it, to prevent or to counteract potential circulatory collapse. Pregnant patients should be placed on their left side [5]. Once supine, patients should not be allowed to sit up until clearly fully stabilized, owing to the risk of ‘empty ventricle syndrome’ which can precipitate a profound loss of blood pressure and death [20].

As mentioned earlier, patients with asthma, particularly those with poorly controlled asthma, are at increased risk of a fatal reaction. In these patients, anaphylaxis may be mistaken for an asthma exacerbation and inappropriately treated solely with asthma inhalers. Therefore, if there are ongoing asthma symptoms in an individual with known anaphylaxis, epinephrine should be given [16].

Supportive therapy such as inhaled beta_2_-agonists (for patients experiencing bronchospasm) and antihistamines (for control of cutaneous symptoms) can also be helpful, but should never replace epinephrine as first-line therapy. Oxygen therapy should also be considered in any patient with symptoms of anaphylaxis, particularly for those with prolonged reactions. Intravenous crystalloid solutions should also be provided since massive fluid shifts can occur rapidly in anaphylaxis due to increased vascular permeability. Volume replacement is particularly important for patients who have persistent hypotension despite epinephrine injections. Vasopressors, such as dopamine, can also be considered if epinephrine injections and volume expansion with intravenous fluids fail to alleviate hypotension. Corticosteroids have a slow onset of action and, therefore, these agents have not been shown to be effective for the acute treatment of anaphylaxis. Theoretically, however, they may prevent biphasic or protracted reactions and, hence, are often given on an empirical basis. To date, there is no conclusive evidence that the administration of corticosteroids prevents a biphasic response [5]. In fact, a recent non-randomized study suggested a number needed to treat (NNT) of 173–176 to prevent a biphasic reaction [21].

If anaphylaxis fails to respond to intramuscular epinephrine and intravenous fluids, an intravenous infusion of epinephrine may be required; however, these infusions should be given by a physician who is trained and experienced in its use and has the capacity for continuous blood pressure and cardiac monitoring.

**Fig. 1:**
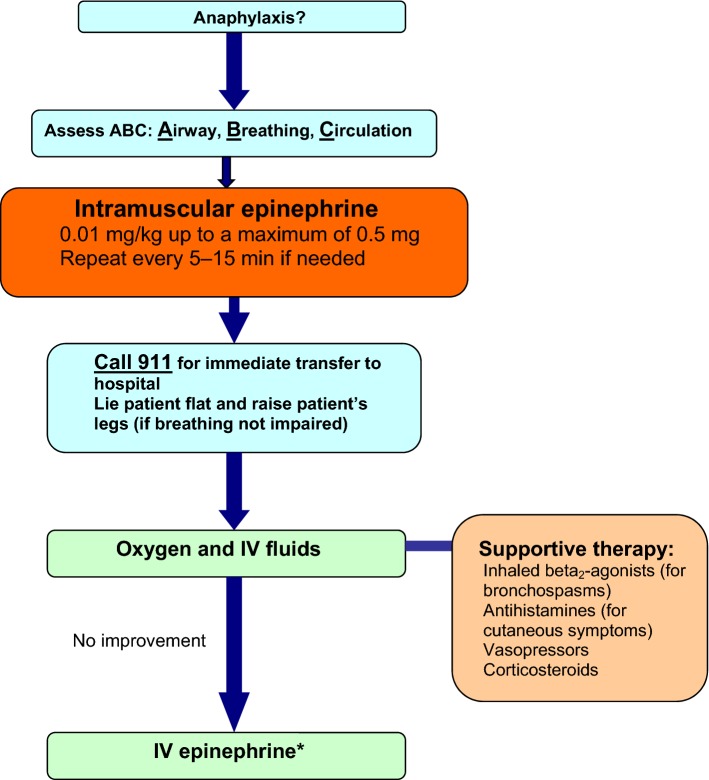
**Simplified algorithm for the acute management of anaphylaxis**. *IV* intravenous. *Should be given by a physician trained in the use of IV epinephrine with capacity for continuous blood pressure and cardiac monitoring

Figure 1 provides a simplified algorithm for the acute management of anaphylaxis.

Following acute treatment, patients should be observed for a period of time due to the risk of a biphasic response or possible recurrence of the reaction as epinephrine wears off. The observation period should be individualized based on the severity of the initial reaction and access to care. Experts have recommended observing patients for 4–6 h following an anaphylactic reaction, with prolonged observation times for patients with severe or refractory symptoms [16].

## Long-term management

The mainstays of long-term management for patients who have experienced anaphylaxis include: specialist assessment, a prescription for an epinephrine auto-injector, patient and caregiver education on avoidance measures, and the provision of an individualized anaphylaxis action plan.

### Specialist assessment

After acute anaphylaxis, patients should be assessed for their future risk of anaphylaxis, ideally by an allergist. These specialists are experienced in identifying and confirming the cause of anaphylaxis, educating patients on appropriate avoidance strategies, drafting an anaphylaxis action plan, and advising whether immunotherapy is appropriate [5, 16].

### Prescription for an epinephrine auto-injector

A prescription for an epinephrine auto-injector should be provided to all patients who have experienced anaphylaxis previously, including those who have had*** any*** rapid-onset systemic allergic reaction (gastrointestinal, respiratory, cardiac); diffuse hives to any food or insect stings; or ***any*** rapid-onset (i.e., minutes to hours) reaction of any severity to the highest risk foods such as peanut, tree nuts, fish, and shellfish [16].

EpiPen^®^ is currently the only epinephrine auto-injector available in Canada, although the Taro-Epinephrine Auto Injector^®^ has been approved by Health Canada but is not yet available. Both products come in two dosages (0.15 and 0.30 mg), which are prescribed according to weight. The 0.30 mg dosage should be used for those weighing 30 kg or more, and the 0.15 mg dosage for children weighing between 15 and 30 kg. Certain sources recommend switching to the 0.30 mg dose at 25 kg rather than 30 kg [22]. These devices should be stored properly (avoiding temperature extremes) and replaced before the expiration date.

Upon prescription of an epinephrine auto-injector, healthcare providers must instruct the patient on how and when to use the device. Instructions on proper use should be reviewed verbally and accompanied by website links and/or written material, and should be reinforced annually.

The currently available epinephrine auto-injectors have needle lengths of approximately 13 and 15 mm for the 0.15 and 0.30 mg doses, respectively. Recent studies suggest that, at these needle lengths, children weighing less than 15 kg are at increased risk of injection into the bone [23] and adult females are at increased risk of subcutaneous injection [24]. Therefore, special counseling on appropriate epinephrine administration in these patients may be needed.

### Avoidance/recognition of co-factors for increased anaphylaxis risk

Patients should be educated on certain co-factors that can lead to an increasingly severe anaphylactic reaction. Exercise can significantly increase the likelihood and/or severity of allergic reactions. Other significant co-factors include: acetylsalicylic acid (ASA)/NSAID use, alcohol use, menses, and concomitant viral illnesses [25].

### Education on avoidance measures

Patients and their caregivers should be educated about agents or exposures that may place them at risk for future reactions, and should be counselled on avoidance measures that may be used to reduce the risk for such exposures. Avoidance strategies should be individualized, taking into consideration factors such as relevant triggers, age, activity, occupation, hobbies, residential conditions, access to medical care, and the patient’s level of personal anxiety. Individuals who have had anaphylactic reactions to foods should be instructed to read food labels carefully, watching for hidden ingredients such as “natural flavour” or “spices” that may indicate the presence of allergens (e.g., peanut, tree nuts, milk, egg, shellfish, fish, sesame, soy and wheat), as well as “may contain” warnings [5]. Recent evidence suggests that peanut allergic children can be desensitized to peanut by feeding them increasing amounts of peanut under close supervision [26]. Similar results have been noted for egg and milk allergy. Although these results are promising, further confirmatory studies in this area are needed before routinely recommending desensitization procedures to patients with these food allergies (for more information, see * IgE-Mediated Food Allergy * and *Non-IgE-Mediated Food Hypersensitivity* articles in this supplement).

Patients with anaphylaxis to medications should be informed about all cross-reacting medications that should be avoided. Should there be a future essential indication for use of the medication causing anaphylactic reactions, it may be helpful to educate patients about possible management options, such as medication pretreatment and use of low osmolarity agents in patients with a history of reactions to radiographic contrast media, or induction of drug tolerance procedures (also known as drug desensitization) [5]. Induction of drug tolerance procedures temporarily modify a patient’s immunologic or non-immunologic response to a drug through the administration of incremental doses of the drug. However, drug tolerance is usually maintained only as long as the drug is administered; therefore, the procedure needs to be repeated in the future if the patient requires the drug again after finishing a prior therapeutic course (for more information, see* Drug Allergy* article in this supplement).

Patients who have had an anaphylactic reaction to an insect sting should be advised about avoidance measures to reduce the risk of future stings. Such measures include: being alert when eating outdoors (as wasps are attracted to food), wearing shoes and long pants when in fields, and having nests or hives near the patient’s home removed [15]. More importantly, however, patients who have previously experienced venom-induced anaphylaxis are often candidates for venom immunotherapy, which is successful in preventing anaphylaxis in up to 98% of patients (see *Allergen-specific Immunotherapy* article in this supplement), and all should be referred for an allergy assessment.

Patients should also obtain and wear medical identification (such as a MedicAlert® bracelet/necklace) that indicates that they have experienced anaphylaxis as well as the responsible agent. Patients should also be instructed to avoid drugs that might increase their susceptibility and/or complicate the management of an anaphylactic event, such as beta-blockers or ACE inhibitors [5].

### Anaphylaxis action plan

A comprehensive, individualized anaphylaxis action plan should be prepared which defines roles and responsibilities and emergency protocols. Important information that should be included in this plan is shown in Table 4 [16, 27]. Examples of such a plan, along with other relevant information and materials, can be downloaded at Food Allergy Canada (www.foodallergycanada.ca) or Food Allergy Research and Education (www.foodallergy.org; a US-based association). Action plans should be reviewed annually and updated if necessary. A copy of the plan should be made available to all relevant persons, such as day-care providers, teachers, and employers. Recommendations for the management of anaphylaxis in schools and other community settings [28] are available through Food Allergy Canada (www.foodallergycanada.ca) or the Canadian Society of Allergy and Clinical Immunology (CSACI) (see http://csaci.ca/patient-school-resources).

**Table 4:** Components of an anaphylaxis action plan [16, 27]

**Contact details** • Names and contact details for emergencies, including family members, allergist/immunologist and family doctor • Contact details for local emergency or ambulance services
**Allergens/triggers** • Clear identification of allergens/triggers to be avoided – Include generic and proprietary names of drugs and possible cross-sensitivities, if relevant
**How to recognize the signs and symptoms of anaphylaxis** • Mouth: itching, swelling of lips/tongue • Throat: itching, tightness, closure, hoarseness • Skin: itching, hives, eczema, swelling, flushing • Gut: vomiting, diarrhea, abdominal pain • Lung: shortness of breath, cough, wheeze • Heart: hypotension, dizziness, syncope, tachycardia • Neuro (or head): lightheadedness • Other: feeling of impending doom, anxiety
**Medications prescribed and when they should be used** • Epinephrine auto-injectors (first-line); should include detailed instructions (with photographs, if possible) on how to correctly administer the auto-injector device (for daycare, school and/or office staff) • Antihistamines (for cutaneous symptoms) • Inhaled beta_2_-agonists (for bronchospasm)
**Where medication is stored at home, work or school**

## Conclusions

Anaphylaxis is an acute, potentially fatal systemic reaction with varied mechanisms and clinical presentations. Prompt recognition and treatment of anaphylaxis are imperative; however, both patients and healthcare professionals often fail to recognize and diagnose anaphylaxis in its early stages. Diagnostic criteria which take into account the variable clinical manifestations of anaphylaxis are now available and can assist healthcare providers in the early recognition of the condition. Immediate intramuscular administration of epinephrine into the anterolateral thigh is first-line therapy for anaphylaxis. Acute management may also involve oxygen therapy, intravenous fluids, and adjunctive therapies such as antihistamines or inhaled beta_2_-agonists. The mainstays of long-term management include specialist assessment, a prescription for an epinephrine auto-injector, patient and caregiver education on avoidance measures, and the provision of an individualized anaphylaxis action plan.

## Key take-home messages

- Anaphylaxis is the most severe form of an allergic reaction that is rapid in onset and potentially fatal.
- Prompt recognition and treatment are critical in anaphylaxis.
- The diagnosis is based primarily on clinical signs and symptoms.
- The most common clinical manifestations are cutaneous symptoms, including urticaria and angioedema, erythema, and pruritus.
- Referral to an allergist or immunologist should be considered for all persons who have experienced a previous anaphylactic episode.
- Epinephrine is the drug of choice for anaphylaxis and should be given immediately, even if the diagnosis is uncertain; intramuscular administration into the anterolateral thigh is recommended.
- There are no absolute contraindications to the use of epinephrine.
- Up to 15% of anaphylaxis cases will have a biphasic response, with a second wave of symptomatology.
- The mainstays of long-term treatment include: specialist assessment, avoidance measures, the provision of an epinephrine auto-injector and an individualized anaphylaxis action plan.

## Abbreviations

IgE: immunoglobulin E
NSAID: non-steroidal anti-inflammatory drugs
ACE: angiotensin-converting enzyme
ASA: acetylsalicylic acid
NNT: number needed to treat
CSACI: Canadian Society of Allergy and Clinical Immunology
